# An improved bacterial mRNA enrichment strategy in dual RNA sequencing to unveil the dynamics of plant-bacterial interactions

**DOI:** 10.1186/s13007-024-01227-x

**Published:** 2024-07-01

**Authors:** Jayabalan Shilpha, Junesung Lee, Ji-Su Kwon, Hyun-Ah Lee, Jae-Young Nam, Hakgi Jang, Won-Hee Kang

**Affiliations:** 1 Department of Horticulture, Division of Applied Life Science (BK21 Four Program), Institute of Agriculture & Life Science, Gyeongsang National University, Jinju, 52828 Republic of Korea; 2 Division of Smart Horticulture, Yonam College, Cheonan, 31005 Republic of Korea

**Keywords:** *Capsicum annuum*, Dual RNA-seq, Gene ontology, Plant-bacterial interactions, Poly A selection, rRNA depletion

## Abstract

**Background:**

Dual RNA sequencing is a powerful tool that enables a comprehensive understanding of the molecular dynamics underlying plant-microbe interactions. RNA sequencing (RNA-seq) poses technical hurdles in the transcriptional analysis of plant-bacterial interactions, especially in bacterial transcriptomics, owing to the presence of abundant ribosomal RNA (rRNA), which potentially limits the coverage of essential transcripts. Therefore, to achieve cost-effective and comprehensive sequencing of the bacterial transcriptome, it is imperative to devise efficient methods for eliminating rRNA and enhancing the proportion of bacterial mRNA. In this study, we modified a strand-specific dual RNA-seq method with the goal of enriching the proportion of bacterial mRNA in the bacteria-infected plant samples. The enriched method involved the sequential separation of plant mRNA by poly A selection and rRNA removal for bacterial mRNA enrichment followed by strand specific RNA-seq library preparation steps. We assessed the efficiency of the enriched method in comparison to the conventional method by employing various plant-bacterial interactions, including both host and non-host resistance interactions with pathogenic bacteria, as well as an interaction with a beneficial rhizosphere associated bacteria using pepper and tomato plants respectively.

**Results:**

In all cases of plant-bacterial interactions examined, an increase in mapping efficiency was observed with the enriched method although it produced a lower read count. Especially in the compatible interaction with *Xanthmonas campestris* pv. *Vesicatoria* race 3 (Xcv3), the enriched method enhanced the mapping ratio of Xcv3*-*infected pepper samples to its own genome (15.09%; 1.45-fold increase) and the CDS (8.92%; 1.49-fold increase). The enriched method consistently displayed a greater number of differentially expressed genes (DEGs) than the conventional RNA-seq method at all fold change threshold levels investigated, notably during the early stages of Xcv3 infection in peppers. The Gene Ontology (GO) enrichment analysis revealed that the DEGs were predominantly enriched in proteolysis, kinase, serine type endopeptidase and heme binding activities.

**Conclusion:**

The enriched method demonstrated in this study will serve as a suitable alternative to the existing RNA-seq method to enrich bacterial mRNA and provide novel insights into the intricate transcriptomic alterations within the plant-bacterial interplay.

**Supplementary Information:**

The online version contains supplementary material available at 10.1186/s13007-024-01227-x.

## Background

Functional genomics and the ease of access to next-generation sequencing (NGS) techniques have brought about a profound revolution in the field of gene expression research [1]. Over the past decade, there has been a growing preference for high throughput RNA sequencing (RNA-seq) technology in transcriptome research over conventional microarray technology [2, 3]. This preference is driven by its enhanced ability to precisely quantify gene expression levels, offering greater sensitivity and specificity in both eukaryotic and prokaryotic organisms [4–8]. Besides the potential for achieving complete genome coverage, RNA-seq offers various advantages over microarray and tag-based methods. These include an unlimited dynamic range, reduced bias, independence from annotations, and the absence of issues related to probe design or cross-hybridization [9, 10]. RNA-seq can distinguish between different mRNA isoforms and non-coding RNAs (ncRNAs), as well as detect splice junctions and precisely locate transcript boundaries [11–14]. In spite of these advantages, RNA-seq for the transcriptional analysis of plant-microbe interactions remains technically challenging, particularly with the microbial transcriptomics. Employing the transcriptomic approach in bacterial cells presents a significant hurdle owing to the considerable abundance of ribosomal RNA (rRNAs), which make up more than 95% of the entire cellular RNA content [15]. As a result, the effective coverage of valuable transcripts is significantly diminished. Hence, achieving cost-effective and comprehensive sequencing of the bacterial transcriptome necessitates the formulation of effective methods for depleting the abundant bacterial rRNA molecules viz., 5 S, 16 S, and 23 S rRNA [16].

Currently, three primary approaches are employed for rRNA depletion. The first uses oligonucleotide hybridization, followed by magnetic bead separation [17]. The second employs complementary oligonucleotide annealing and enzymatic digestion of duplex hybrids using specific nucleases [18]. The third restricts cDNA synthesis to non-rRNA templates through a specialized primer mixture during cDNA synthesis, excluding rRNA matches [19]. Numerous commercially available kits have been used for eliminating bacterial rRNA from total RNA samples. These encompass the MICROBExpress bacterial mRNA enrichment kit from Thermo Fisher Scientific, the MICROBEnrich™ kit, from Thermo Fisher Scientific and Ambion, the RiboMinus transcriptome isolation kit for bacteria from Thermo Fisher Scientific, NEBNext bacteria rRNA depletion kit from New England Biolabs, mRNA-ONLY prokaryotic mRNA isolation Kit from Epicentre Biotechnologies, Terminator™ kit from Lucigen, Ribo-Zero rRNA depletion kit from Illumina and Epicentre and riboPOOLs from siTOOLs [20, 21]. Among them, the mRNA-ONLY and Terminator kits operate based on the concept of utilizing 5´-monophosphate-dependent exonuclease to break down the processed RNA molecules with 5’-phosphorylation, like rRNAs. However, this approach has exhibited lower effectiveness compared to alternative rRNA depletion methods, resulting in only a moderate enhancement (1.9 to 5.7-fold) of bacterial mRNA enrichment, with fewer than 25% of aligned sequencing reads corresponding to transcripts other than rRNA [22]. The MICROBEnrich™ kit utilizes an innovative magnetic bead capture hybridization technique to effectively remove mammalian RNA (including human, mouse, or rat RNA) from intricate mixtures of mammalian host-bacterial RNA [23, 24]. This method eliminates over 90% of mammalian RNA by concomitantly eliminating polyadenylated mRNAs along with 18 S rRNA and 28 S rRNA. Nevertheless, MICROBEnrich does not eliminate small RNAs like 5 S rRNA and tRNA from the enriched bacterial RNA pool. Therefore, it necessitates the utilization of additional kits to eliminate small RNAs before initiating the MICROBEnrich procedure. Similarly, the MICROBExpress and RiboMinus kits target oligonucleotides corresponding to 16 S and 23 S rRNA but do not eliminate 5 S rRNA. In contrast, Ribo-Zero kit captures 5 S rRNA in addition to 16 S and 23 S rRNA by magnetic beads. Unlike MICROBEnrich, Ribo-Zero kits do not simultaneously eliminate poly A mRNA. Instead, they selectively deplete rRNA from total RNA samples, allowing for a more comprehensive examination of the non-rRNA portion of the transcriptome. Many studies have demonstrated the effectiveness of the Ribo-Zero kit in eliminating rRNA preceding the RNA-seq library construction [16, 20, 23–25]. Thus, employing Ribo zero kits along with poly A mRNA selection represents an optimal tactic to improve dual RNA sequencing of host-bacterial samples, as demonstrated in this study.

Comprehending the intricate interaction between infecting bacteria and their eukaryotic host is crucial for unraveling the pathogenesis and advancement of diseases as well as their persistence within host cells. Through the concurrent capture of active genes from both the bacteria and the host, dual RNA-seq offers a comprehensive overview of the molecular dynamics involved in bacterial infection processes and the corresponding host responses. However, the profiling of *in planta* bacterial transcriptomes in dual RNA-seq presents several challenges. For instance, bacterial RNAs might comprise less than 1% of the total RNA content, whereas plant rRNA can make up as much as 98% of the total RNA within an infected cell. Particularly, acquiring sufficient bacterial RNA during early stages of infection remains a notable challenge [26]. Compared to standard transcriptomic investigations, dual RNA sequencing introduces technical hurdles arising from the varying proportion of reads originating from the less abundant (bacterial) transcripts in contrast to the more abundant (plant) transcripts, along with the substantial difference in total RNA content where the host significantly outnumbers the bacterial counterpart [15]. Therefore, along with the removal of rRNA, it is imperative to implement mRNA enrichment approaches to guarantee cost-effective sequencing of an ample amount of non-rRNA sequences [16]. Prokaryotic mRNAs are relatively less stable and lack a 3’ polyadenylated tail, hindering hybridization capture, cDNA synthesis, and poly (T) oligomer amplification [27]. Conversely, eukaryotic mRNAs are stable, contain elongated poly (A) tails, and can be enriched using oligo (dT) primers, effectively isolating them from rRNA molecules for sequencing library construction. PCR amplification plays a crucial role in well-known RNA-seq library construction methods such as TruSeq, in enhancing the concentration of cDNA prior to sequencing [28]. Nonetheless, there remains an uncertainty regarding the impact of PCR on the mapping ratio to the genome and coding sequences (CDS), as well as the extent to which PCR amplification introduces noises, potentially diminishing the accuracy of transcript quantification.

Several pathogenic bacteria affect a wide range of crops worldwide, causing considerable damage to crop output and consequently significant financial losses. Bacterial leaf spot is one of the common and devastating diseases in pepper (*Capsicum annuum* L.) and tomato (*Solanum lycopersicum* L.) caused by *Xanthomonas campestris* pv. *vesicatoria* (Xcv) leading to significant defoliation, decreased plant vitality and substantial reductions in crop yield [29, 30]. Generally, host-pathogen interactions can be categorized into two primary categories: (1) a compatible interaction, which lead to a successful infection and subsequent disease (2) an incompatible interaction, where the pathogen is successfully defeated by the plant’s defense mechanisms. In case of an incompatible interaction, as a resistance measure, plants trigger a hypersensitive response (HR), which is a cellular death response that occurs at the entry point of the pathogen, effectively halting the further advancement of the pathogen [31]. The HR responses caused by *Xanthomonas axonopodis* pv. *glycines* (Xag) is an example for an incompatible non-host interaction in pepper plants [32]. On the other hand there are several rhizosphere associated bacteria which mostly exert neutral effect or positive effect on the growth and wellbeing of their host plants through complex interactions [33]. *Flavobacterium dauae*, is one such bacterial strain isolated from the rhizosphere soil of tomato [34]. The understanding of various plant-bacterial interactions will aid in the development of innovative strategies for managing bacterial diseases. Thus, the documentation of transcriptomic profiles of both the host and bacteria at various time points following infection could facilitate the examination of the molecular changes driving host responses and bacterial adaptability. In the present investigation, we have devised a high throughput dual RNA sequencing method involving mRNA enrichment strategies such as rRNA depletion combined with poly A enrichment using Dynabeads, to investigate the molecular dynamics involved in host-bacterial interactions using tomato and pepper plants. Moreover, the effect of PCR cycles on the mapping ratio of plant-bacterial samples to the genome and CDS of bacteria has been examined.

## Materials and methods

### Plant materials and bacterial inoculation

To assess the host-bacterial interactions, the tomato cultivar ‘Heinz’ was inoculated with *Flavobacterium dauae*, a beneficial rhizosphere associated bacteria. Hot pepper cultivars, ‘ECW30R’, and ‘Bukang’ were inoculated with *Xanthomonas campestris* pv. *vesicatoria* race 3 (Xcv3) showing host resistant response and *Xanthomonas axonopodis* pv. *glycines* 8ra (Xag8ra) showing non-host resistant response. The *F. dauae* was cultured in Trypticase Soy Agar (TSA) media at 30^o^C for 2 days and subsequently diluted to a concentration of 10^9^ CFU/mL in Hoagland solution (Sigma-Aldrich, St. Louis, CA, USA). The *F. dauae* suspension was inoculated into 3 days-post-germination-stage of tomato seedlings. Then, *F. dauae* inoculated plants were grown in a growth chamber at 28^o^C and 16-h light / 8-h dark photoperiod, and inoculated seedlings were harvested at 48 h post inoculation (hpi). For the preparation of Xcv3 and Xag8ra inocula, we followed the methods as described previously [31, 35]. Xcv3 and Xag8ra were cultured in Luria-Bertani (LB) media containing rifampicin (50 mg/L) for 2 days and then were suspended in 10 mM MgCl_2_ and diluted to a final concentration of 10^8^ CFU/mL. Subsequently, Xcv3 and Xag8ra suspensions were infiltrated into the leaves of 6 true-leaf-stage of ‘ECW30R’ and ‘Bukang’, respectively. The leaves of Xcv3 inoculated pepper plants were harvested at 12, 24, and 48 hpi, whereas Xag8ra inoculated plants were sampled at 48 hpi. All bacterial inoculation was performed in at least three independent experiments with 12–16 plants for each experiment. The pathogen control for Xcv3 was prepared using a suspension in 10 mM MgCl_2_ at a concentration of 10^8^ CFU/mL.

### RNA extraction and RNA-seq library construction

Total RNA was extracted from the leaves of pepper and roots of tomato seedlings (100 mg) using Trizol reagent (Invitrogen, Carlsbad, CA, USA) following the manufacturer’s instructions. RNA-seq libraries were prepared from the five microgram of RNA using TruSeq kit (Illumina, San Diego, CA, USA; hereafter conventional method) and modified strand-specific method (hereafter enriched method). For the conventional method, we performed the construction of RNA-seq libraries using TruSeq prepration kit with ribo depletion following the manufacturer’s recommendations. For the enriched method, we used a modified strand-specific library construction method [36–38]. To isolate the poly A RNA, the RNA samples were treated with Dynabeads™ oligo(dT)_25_ (Invitrogen, Carlsbad, CA, USA) and collected separately. In the conventional method, 1X volume (15 μL) of Dynabeads is used while for the enriched method, 3.3X (50 μL) of Dynabeads are employed, denoted as B1X and B3.3X respectively. The 1X volume of Dynabeads was also tested for the enriched method as the reference. For the mock controls (*C. annuum* and Xcv3), the conventional RNA-seq method was applied with 1X concentration of Dynabeads being used for *C. annuum* and no Dynabeads added for Xcv3 (Table 1). The remaining RNA samples without Dynabeads™ oligo(dT)_25_ were subjected to ribo depletion by Ribo-Zero Plant Kit (Illumina, San Diego, CA, USA) to remove rRNAs from both plant and bacteria in the proportion of 95:5 according to host vs. bacteria ratio in infected samples. Subsequently, the rRNA removed and poly A removed RNA samples were pooled together and progressed to cDNA synthesis. The sequential steps for library construction were carried out following the strand-specific library method as previously described [36–38]. Further, to optimize the enriched method 10, 12, 15, or 20 cycles of PCR enrichment were performed to prepare the libraries. The quality and size (approximately 200–400 bp in size) of all libraries were evaluated with Agilent 2100 Bioanalyzer (Agilent Technologies, Inc., Santa Clara, CA, USA).

**Table 1:** Statistical summary and comparative read mapping of RNA-seq data used in the study

Plant	Bacteria	Type of pant-bacterial interaction	Method	Time point (hpi)	Modification^a^	Clean reads^b^	Plant		Bacteria			
							Mapped genome^c^	Mapped genome ratio^d^ (%)	Mapped genome^c^	Mapped genome ratio^d^ (%)	Mapped CDS^e^	Mapped CDS ratio^f^ (%)
*S. lycopersicum*	*F. dauae*	Positive	Conventional	48	B1X/C10	119,836,355	114,930,404	95.91	1,434,056	1.2	183,600	0.15
			Enriched	48	B1X/C10	12,299,298	11,505,248	92.3	127,753	1.1	70,554	0.57
					B3.3X/C10	18,368,732	17,565,053	95.41	353,341	1.94	182,212	0.99
					B3.3X/C15	16,216,836	15,577,275	94.98	273,359	1.8	149,081	0.9
					B3.3X/C20	13,658,775	13,095,302	95.47	205,142	1.55	109,220	0.8
*C. annuum*	Xag8ra	Incompatible, non-host resistance	Enriched	48	B3.3X/C15	23,631,656	21,521,157	91.07	212,168	0.90	144,087	0.61
					B3.3X/C20	146,198,300	131,961,491	90.59	1,475,467	0.94	1,007,895	0.64
*C. annuum*	Xcv3	Incompatible, host resistance	Enriched	24	B3.3X/C10	26,886,048	24,293,063	90.47	496,519	1.81	332,082	1.21
					B3.3X/C15	23,797,803	21,503,984	90.31	507,576	2.16	347,292	1.48
					B3.3X/C20	45,286,688	40,699,396	89.82	1,143,759	2.55	846,593	1.89
*C. annuum*	Xcv3	Incompatible, host resistance	Enriched	12	B3.3X/C12	26,275,757	22,468,782	85.6	194,644	0.74	103,713	0.39
				24	B3.3X/C12	24,281,467	21,360,839	88.03	509,365	2.12	317,319	1.32
				48	B3.3X/C12	24,790,157	18,854,536	76.17	3,781,607	15.09	2,249,053	8.92
			Conventional	12	B1X/C10	30,157,170	27,458,323	91.03	159,407	0.53	99,825	0.33
				24	B1X/C10	34,311,587	31,031,802	90.44	494,298	1.44	290,749	0.85
				48	B1X/C10	30,267,293	24,208,517	80.01	3,152,686	10.4	1,818,018	5.99
-	Xcv3 (Mock control)	-	Conventional	0	B0X/C10	32,050,429	-	-	30,671,378	95.69	22,184,190	69.17
*C. annuum* (Mock control)	-	-	Conventional	0	B1X/C10	30,610,179	27,162,689	88.7	-	-	-	-

^a^ B1X, 1X volume of Dynabeads; B3.3X, 3.3X volume of Dynabeads and B0X, not addition of Dynabeads; C (10, 12, 15 and 20), number of PCR cycles in PCR enrichment step

^b^ Trimmed reads with the mean of two or three replicates

^c^ Number of reads (mean of two or three replicates) mapped to the plant or bacterial genome

^d^ Percentage of mapped reads (average of two or three replicates)

^e^ Number of reads (mean of two or three replicates) mapped to the bacterial coding sequences (CDS)

^f^ Percentage of mapped reads to the bacterial CDS (average of two or three replicates)

### Sequencing and data processing

The libraries were sequenced using a 101-nt, paired-end module on a HiSeq 2000 platform (Illumina, San Diego, CA, USA). The low quality reads and adapter sequences were filtered and trimmed using Cutadapt [39] and Trimmomatic [40]. The rRNA filtering was performed using SortMeRNA (version 2.1b) and the silva database [41, 42]. The processed clean reads were aligned to reference genome and gene model of each sample including *F. dauae* TCH3−2 (GCF_004151275.1 ASM415127v1), Xcv3 (GCF_000009165.1 ASM916v1), and Xag8ra (GCF_001854145.2 ASM185414v2) using Hisat v2-2.1.0 software with default values [43]. Hierarchical clustering of transcriptomes for sample validation was performed using the hclust function in R. Raw read counts were normalized using FPKM methods [44]. Differential expressed genes (DEGs) were identified from comparison between Xcv3 treated samples and the pathogen mock control using the DESeq2 [45] package (FDR < 0.05). The database construction for Gene Ontology (GO) enrichment analysis was performed using BLAST2GO [46] for *Xanthomonas* gene functional annotation. Based on the constructed GO database, GO enrichment analysis was performed using GOseq [47]. The GO enrichment results were visualized using ggplot2 R package [48].

### Real-time qRT-PCR measurements

Quantitative reverse transcription PCR (qRT-PCR) assays were conducted with the same total RNA samples that were used for DEG profiling of Xcv3. The real-time qRT-PCR was performed using Rotor Gene Q system (QIAGEN, Hilden, Germany) and PCR primers were designed with Primer3 plus software and the primers used for validation is listed in Table S1. Reverse transcription (RT) of 80 ng total RNA was carried out in a total volume of 20 ul with a Superscript^®^ IV Reverse Transcriptase (Invitrogen, Waltham, MA, USA) using random hexamers. Cycling parameters were initial denaturation at 95℃ for 2 min, then 50 cycles of 95℃ for 20 s, 20 s at primer specific annealing temperatures, and 72℃ for 20 s, followed by a cDNA dissociation program from 72 to 95℃. Each 20 ul reaction mixture contained 8 ul of diluted cDNA (1:5), 2 ul of each primer (10 μm) and 10 ul of 2X QuantiNova ^TM^ SYBR Green master mix (QIAGEN, Hilden, Germany). Serial dilutions of the pooled cDNA samples were used to generate standard curves to acquire the amplification efficiencies of PCR reactions. A bacteria specific gene encoding *lepA* was selected as the reference gene [49]. We normalized the expression of the target gene using housekeeping genes and also carried out normalization based on Xcv3-12 hpi cDNA samples to measure the relative expression value. Our preliminary evaluation indicated that expression of *lepA* displays little variation across all samples tested. All real-time qRT-PCR assays were carried out in three independent parallel experiments.

## Results

### Experimental design for the enrichment of bacterial mRNA in plant-bacterial sample

**Fig. 1:**
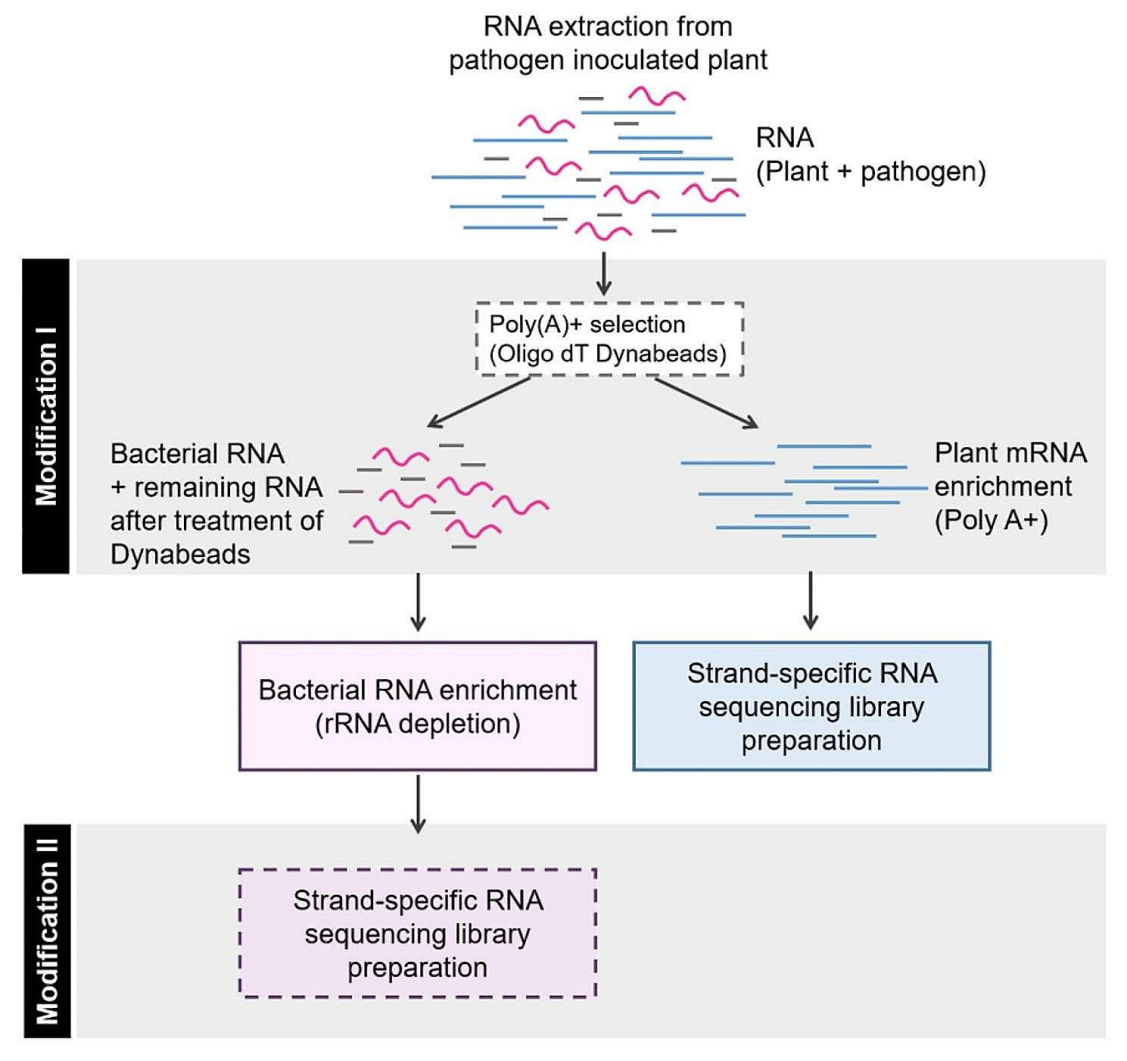
An overview of modified workflow for bacterial mRNA enrichment in dual RNA sequencing library preparation method

In the present study, in order to perform the enrichment of bacterial RNAs within plant samples infected by bacteria, and subsequently facilitate transcriptome analysis, we have introduced modifications to two key steps within the strand-specific RNA sequencing (ssRNA-seq) library preparation protocol, as illustrated in Fig. 1. The initial step pertains to the removal of rRNA. In our efforts to boost the bacterial mRNA yield, we undertook a dual approach aimed at concomitantly eliminating rRNA. This involved employing both the mRNA enrichment method through the subtraction of plant poly (A) using Dynabeads and rRNA depletion for both bacterial and plant components. Following the removal of poly A and depletion of rRNA, the resultant samples were employed in the construction of RNA-seq libraries. To further enhance the enrichment of bacterial mRNA, a second modification was implemented during the PCR enrichment step within the ssRNA sequencing library preparation process (Fig. 1). By varying the number of PCR cycles, we systematically enriched the entire sample, including the bacterial mRNA. Consequently, we conducted experiments to assess and optimize the enrichment of bacterial mRNAs with different PCR cycle numbers and compared them with the typical PCR cycle number of 10. In our enriched method, we employed an increased concentration of Dynabeads (3.3X) and subsequently assessed its impact in comparison to the standard 1X Dynabeads concentration, which is generally utilized for ssRNA-seq library preparation (Table 1). Overall, we assessed the effectiveness of two RNA-seq approaches: (i) the standard Illumina RNA-seq method (conventional method), which comprises of poly A removal using 1X concentration of Dynabeads, rRNA depletion using Ribozero, and a PCR enrichment step using a constant PCR cycle (10) (ii) the bacterial mRNA enrichment method (enriched method) which involves the subtraction of poly A using 3.3X Dynabeads, rRNA depletion using Ribozero, and a modified PCR enrichment step with varied number of PCR cycles (10, 12, 15, and 20). To effectively capture the distinct transcriptional responses of both bacteria and the host, it is crucial to carefully select time points that correspond to various stages of the infection process. During the initial stages of infection, it is probable that only a small amount of bacterial RNA is present, especially when employing low MOIs (Multiplicities of Infection). Therefore, in this investigation, we conducted transcript analysis of *F. dauae* inoculated tomato roots at 48 hpi, and for Xag8ra and Xcv3 infections in pepper plants, transcript analysis was performed at 12, 24, and 48 hpi employing both conventional and enriched methodologies (Table 1). Including appropriate mock-infected control samples for both the pathogen and the host is crucial for distinguishing between host responses that are specific or nonspecific to the bacterium. Hence, we used mock controls corresponding to the host (*C. annuum*) and the pathogen (Xcv3) in the current study.

### Effect of rRNA depletion on the mapping ratio to the genome and CDS of bacteria

As the maximum depletion of rRNA and retrieval of mRNA reduces sequencing costs, we aimed to deplete rRNA (from both plant and bacteria) and retrieve mRNA through poly A selection simultaneously. A total of 44 RNA-seq libraries were generated including 16 libraries for the conventional method and 28 libraries for the enriched method. Subsequently, the raw RNA sequences were filtered and trimmed after sequencing. The processed reads from the conventional method generated an average of 119 M clean reads, while the enriched method produced approximately 18 M clean reads in *F. dauae* infected tomato samples (B3.3X/C10 in Table 1). Upon aligning the reads to the *F. dauae* genome and its coding sequences (CDS), the conventional method exhibited mapping rates of 1.2% and 0.15% to the genome and CDS respectively. Conversely, the enriched method displayed markedly higher mapping ratios, with 1.94% and 0.99% of reads aligning to the genome and CDS, respectively of *F. dauae*. This represents an increase of approximately 1.62-fold for genome mapping and 6.6-fold for CDS mapping of *F. dauae* compared to the conventional method (Table 1). In the context of Xcv3-infected pepper plants, the conventional method produced clean reads ranging from 30 M to 34 M reads at different time points while the enriched method yielded a wider range of reads, varying from 23 M to 45 M reads across various time points and different PCR cycle numbers. When compared to the conventional method, the enriched method exhibited a substantially higher mapping ratio of reads to the genome and CDS of Xcv3. Notably, at 48 hpi, the enriched method demonstrated a mapping ratio of 15.09% to the genome and 8.92% to the CDS of Xcv3. These values indicated a substantial improvement, with a 1.45-fold increase in genome mapping and a 1.49-fold increase in CDS mapping of Xcv3 compared to the conventional method. Although the enriched method yielded a lower read count than the conventional method, it demonstrated an enhanced mapping ratio. The variation observed in the ratio of clean read count between *F. dauae*-infected tomato and Xcv3-infected pepper samples across the two methods could be attributed to variations in several factors including bacterial load, transcriptome complexity, host response variability, specificity and efficacy of enrichment, as well as experimental and biological variability. Taken together, these results suggest that the modification involving simultaneous rRNA depletion and PCR enrichment in the enriched method leads to an elevated proportion of bacterial mRNA, at a reduced sequencing depth in plant-bacterial samples.

### Effect of modified PCR enrichment step on the mapping ratio and RNA-seq library preparation

**Fig. 2:**
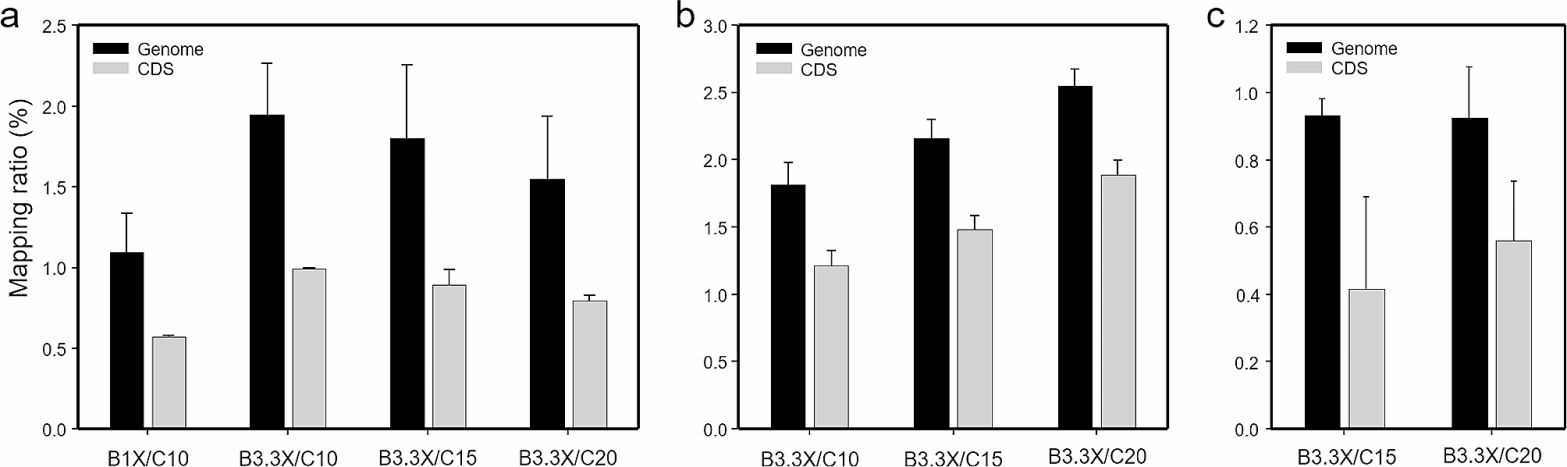
Impact of bacterial mRNA enrichment in Dual RNA-seq on genome and CDS mapping ratio. Ratio of reads aligned with (**a**) genome and CDS of *F. dauae* from *F. dauae* infected tomato at 48 hpi, (**b**) genome and CDS of Xcv3 from Xcv3 infected pepper at 24 hpi, and (**c**) genome and CDS of Xag8ra from Xag8ra inoculated pepper at 48 hpi. B, Dynabeads; 1X, 1X volume of Dynabeads as the original protocol; 3.3X, 3.3X volume of Dynabeads in the enriched protocol; C, number of PCR cycles in PCR enrichment step

To enhance the abundance of bacterial mRNA in the sequencing reads, we adjusted the number of PCR cycles in the enriched method, as compared to the conventional ssRNA-seq library preparation method, which typically utilizes 10 PCR cycles with 5 μg of total RNA. The reads were aligned to bacterial genome and CDS in order to evaluate the increase in bacterial mRNA as the number of PCR cycle increases. Distinct responses were observed in the mapping ratios of *F. dauae* infected tomato as well as Xcv3 and Xag8ra infected hot peppers in relation to variations in PCR cycle numbers. In case of *F. dauae* infected tomato, an inverse relationship was observed between the read mapping ratio and PCR cycles (Fig. 2A). However, in hot peppers infected with Xcv3 and Xag8ra, a positive correlation where an increase in PCR cycle number is accompanied by a corresponding increase in the mapping ratio was observed (Table 1) (Fig. 2B, C). While an increase in PCR cycles improved the mapping ratio of Xcv3 and Xag8ra infected pepper samples, it did not yield the desired library length, as evidenced by the size distribution pattern of cDNA libraries (Fig. S1). Therefore, the variations in mapping ratios at different PCR cycle numbers across different samples could be influenced by several factors that reflect the complex interplay between host-bacterial interactions, including host immune responses, bacterial virulence factors, infection dynamics and host specificity. Among all the examined libraries, the 10 and 12 PCR cycles exhibited a single peak corresponding to the expected library length, thus confirming the precise construction of these libraries. Conversely, the libraries subjected to 15 and 20 PCR cycles displayed either a collapsed peak or a secondary peak of unexpected size, indicating the generation of artifacts due to excessive PCR cycling (Fig. S1). Overall, the findings imply that despite the presence of a limited quantity of bacterial mRNA in 10 or 12 PCR cycles than higher PCR cycle numbers, it was appropriate for the ssRNA-seq library preparation with 5 μg of total RNA in the initial step.

### Mapping and gene expression profile of Xcv3 infected pepper at various time points

**Fig. 3:**
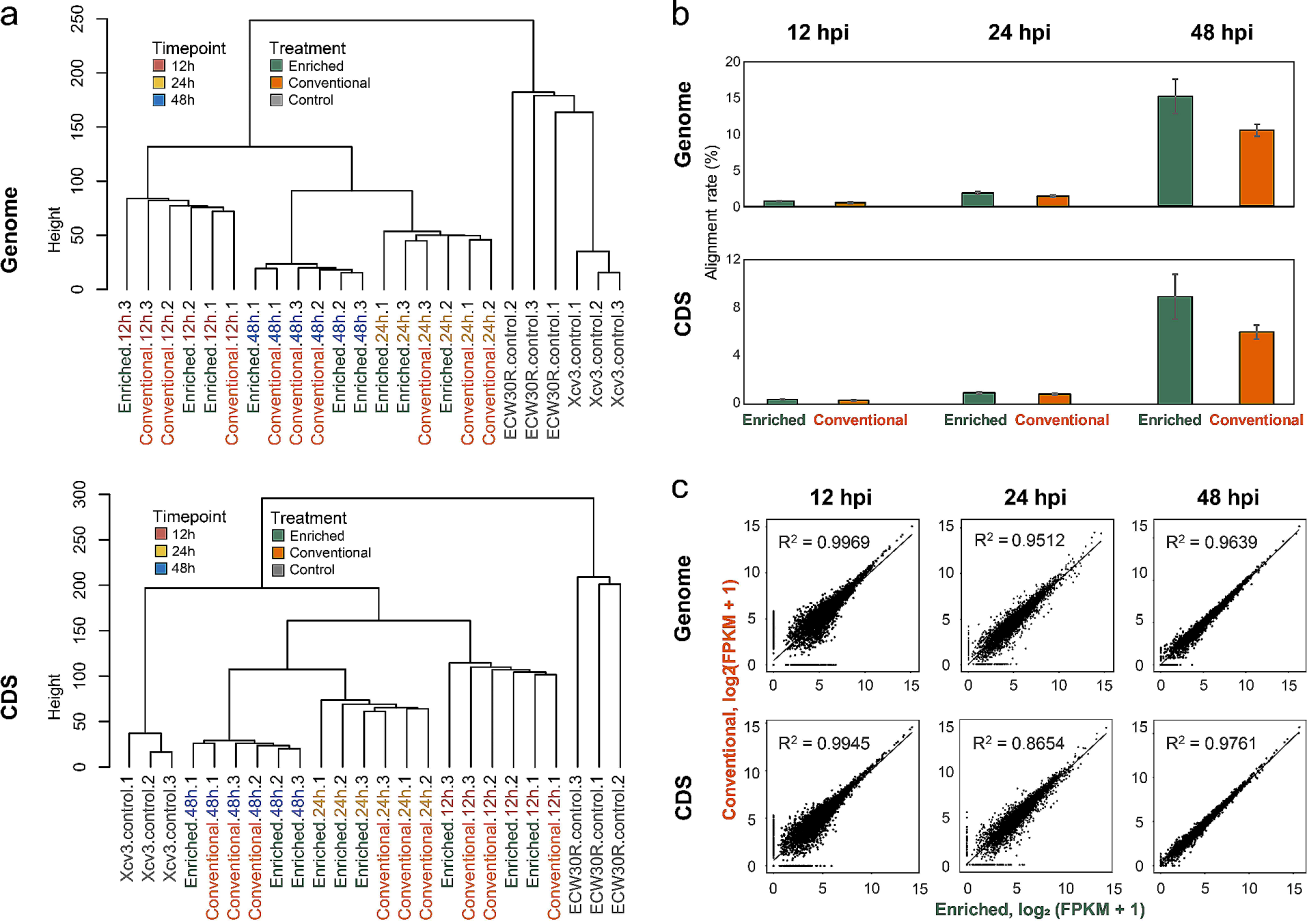
Mapping profiles to genome and CDS of conventional and enriched RNA-seq libraries using Xcv3 transcripts. (**a**) Hierarchical clustering by mapping ratio to pathogen genome and CDS. (**b**) The average rate of aligned reads to reference genome and CDS for each library preparation method at same time points. All data indicate the average of mapping ratio ± SD from three replicates of RNA-seq. (**c**) Pairwise comparison of mean normalized gene expression using all samples for each library preparation method. R^2^, coefficient of determination value

A hierarchical clustering was performed to identify the phylogenetic relationship between the mapping profiles of Xcv3 infected pepper to the genome as well as to the CDS of Xcv3 at various time points (Fig. 3A). The mapping patterns of samples from each time point to the genome of the pathogen were similar and they were grouped together, regardless of the diverse sequencing methods employed. This suggests that the transcript profiles are distinct and characteristic of specific time points. A similar clustering pattern was also noticed when examining the mapping profiles of reads to the CDS of the pathogen among different time points. The mock control samples (ECW30R and Xcv3) exhibited a similar mapping profile to their respective genomes and were grouped together in one cluster. However, their mapping patterns to the CDS differed noticeably, leading to the formation of distinct clusters (Fig. 3A).

The mapping profiles of reads from Xcv3 infected pepper samples to the genome and CDS of Xcv3 varied significantly among various time points (Fig. 3B). The mapping ratio to both the genome and CDS increased as the time of infection increased. The enriched method yielded the highest alignment rates (15.09% and 8.92%), when aligning the reads from samples collected at 48 hpi to the genomic and CDS of the pathogen, respectively. Further, a pairwise comparison of mean normalized gene expression was performed between the conventional and enriched method using all the biological replicates for each time point (Fig. 3C). When examining the gene expression values represented as the fragments per kilo base of transcript per million mapped reads (FPKM) at different time points, it became evident that there was a stronger correlation (R^2^ = 0.99) among the biological replicates from conventional and enriched methods at 12 and 24 hpi, irrespective of genome or CDS region. Relatively, a lower correlation (R^2^ = 0.96; R^2^ = 0.97) in terms of gene expression was observed when comparing samples between the conventional and enriched methods at 48 hpi for mapping to the genome and CDS respectively. Thus, the results indicated that peppers infected with Xcv3 exhibit significant dynamics in the mapping ratio and gene expression profile across various infection time points consistent to both conventional and enriched methods.

### Differential gene expression analysis at various points of Xcv3 infection

**Fig. 4:**
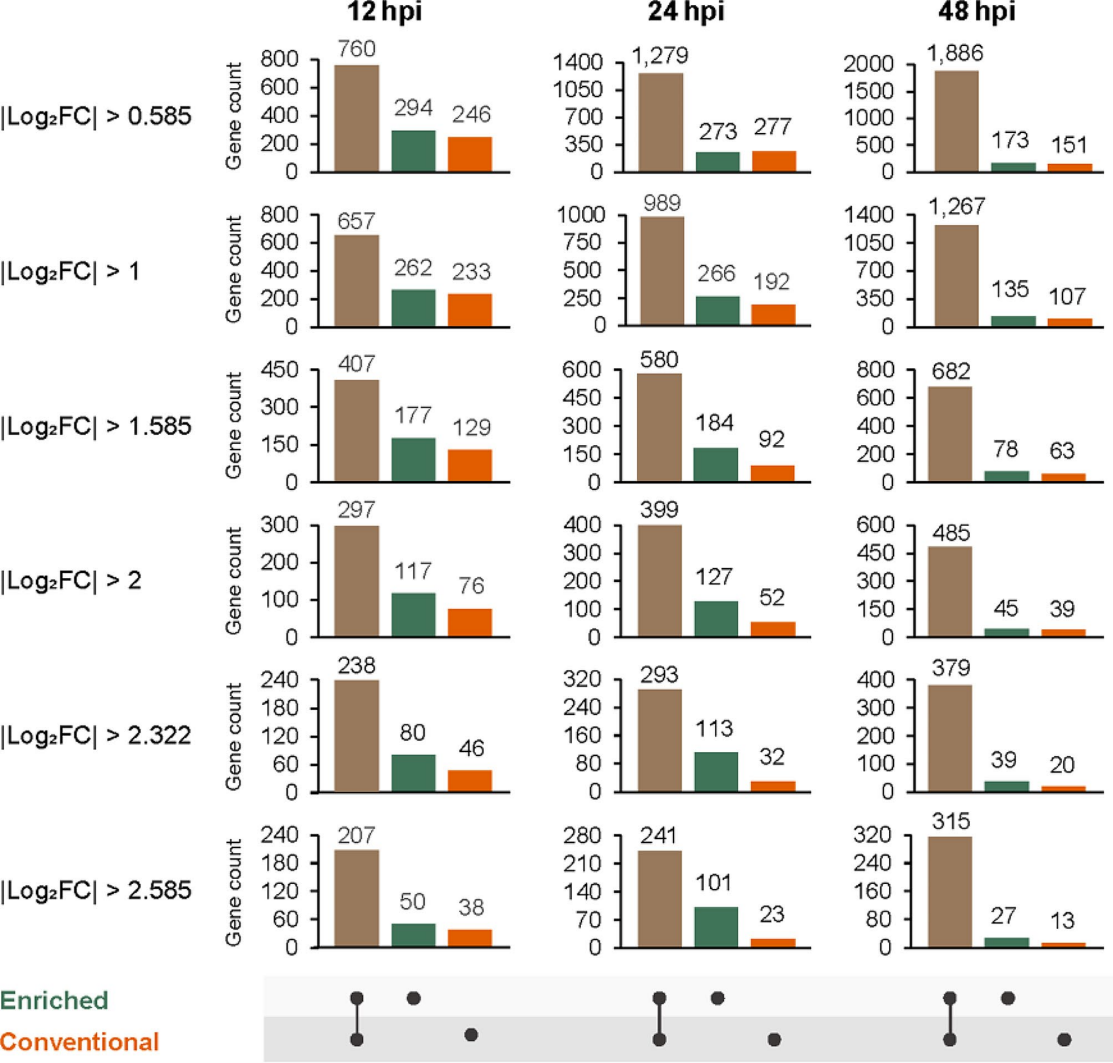
Number of differentially expressed genes (DEGs) in Xcv3 by enriched and conventional RNA-seq library preparation. Graphs in red, green and brown color indicate the number of DEGs identified in conventional, enriched and both methods respectively according to the series of log_2_FC

The differential gene expression analysis in Xcv3 infected pepper plants showed significant differences in Differentially Expressed Genes (DEGs) between conventional and enriched methods at various infection time points. Differences in the number of DEGs were evaluated by comparing the conventional and enriched methods across various fold change (FC) thresholds represented as log_2_ FC (Fig. 4). In both methods, there was a significant upregulation of genes associated with different infection time intervals. The number of genes that are upregulated and downregulated are indicated in Table S2. The enriched method consistently exhibited an elevated number of DEGs than the conventional method at all fold change threshold levels examined at various time points. Among all fold change threshold levels examined, log_2_ FC > 0.5 showed the highest count of DEGs (294) in the enriched method than the conventional method (246) during an early infection time (12 hpi). At the later stage of infection (48 hpi), a significantly reduced number of DEGs were observed in both the methods. However, a larger set of common genes (1,886) between the two methods was identified at 48 hpi at log_2_ FC > 0.5. Overall, in the context of the enriched method, the examination of DEGs at different time points revealed that 24 hpi displayed the highest number of DEGs across all fold change threshold levels except log_2_ FC > 0.5 (Fig. 4). Additionally, to verify this result, among DEG genes, six genes (CAJ23004, CAJ22057, CAJ22060, CAJ22069, CAJ24688, CAJ25348, CAJ21932) were randomly selected, and their expression was confirmed by qRT-PCR (Fig. S2A). In subsequent qRT-PCR analysis, these genes had similar expression patterns showing high induction compared with those in RNA-seq analysis (Fig. S2B).

### Gene ontology enrichment analysis

**Fig. 5:**
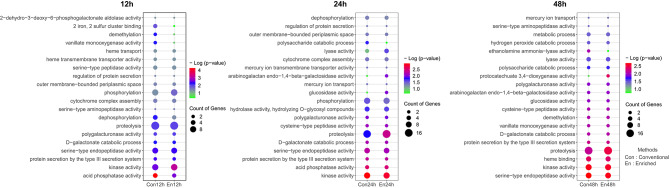
The Gene Ontology (GO) enrichment analysis of *C. annuum* infected by Xcv3. The bubble chart showing the enrichment of DEGs associated with various biological and molecular functions at various infection time points between the conventional and enriched methods. The bubble color indicates -Log (*p*-value) and the bubble size indicates the gene count. The chart displays the top 20 GO terms. The y-axis indicates the GO terms, and the x-axis indicates the -Log (*p*-value). The term ‘Con’ indicates the conventional method while ‘En’ indicates the enriched method

An assessment of the biological functions of all the identified DEGs obtained through both conventional and enriched methods was done using Gene Ontology (GO) enrichment analysis. The bubble plot analysis revealed a significant enrichment of DEGs at various infection time points. Particularly, this enrichment was more pronounced at the 48 hpi using the enriched method (Fig. 5). When examining the top 20 DEGs among the enriched and conventional methods, it became evident that they displayed enrichment in similar GO terms, including various biological processes and molecular functions. In terms of biological processes, DEGs were significantly enriched in proteolysis and protein secretion by the type III secretion system. Thus, the secretion of proteolytic enzymes and translocation of effector proteins could aid the pathogen to invade and cause infection in the host plants. Within molecular function, significant enrichment of DEGs was observed in kinases, serine-type endopeptidases, and heme binding activities. This demonstrates that the enriched method has proven to be equally effective as the conventional method in elucidating gene ontology.

## Discussion

The bacterial spot disease in tomato and pepper plants is attributed to the pathogen Xcv, while Xag is responsible for inducing HR in these plants [50, 51]. The necrotrophic pathogen Xag triggers the development of a bacterial pustule in its host plant, soybean, but does not elicit disease symptoms in non-host plant like hot pepper under natural conditions [30, 32]. Nonetheless, the infiltration of Xag into leaf tissues of non-host plants results in HR, which are associated with the activation of *HR* and pathogenicity genes (*hrp*) [52]. Reports indicate that the pathogen Xcv triggers cell death in pepper plants while inhibiting defense responses in tomato plants [30, 53]. Simultaneous analysis of the gene expression of the two interacting species is valuable for gaining a thorough understanding of plant–bacterial interactions as it can reveal new virulence factors and host response pathways. RNA-seq allows for the simultaneous measurement of expression levels of numerous genes, offering valuable insights into functional pathways and regulatory networks involved in host-pathogen interactions [54, 55]. In the present study, we attempted to evaluate the efficiency of bacterial mRNA enrichment method in dual RNA sequencing involving three types of plant-bacterial interactions such as (i) incompatible host resistant response in Xcv3 infected pepper, (ii) incompatible non-host resistant response in Xag infected pepper and (iii) positive/beneficial response in *F. dauae* infected tomato plants.

In order to facilitate effective transcript and gene detection, it is essential to eliminate highly abundant rRNAs from total RNA prior to sequencing. Common techniques involve the isolation of polyadenylated RNA (poly A) transcripts using oligo (dT) primers and the removal of excessively abundant rRNAs through hybridization capture, followed by separation using magnetic beads. The standard RNA sequencing approaches uses rRNA depletion alone or in combination with poly A selection depending upon the specific goal of the experiment [56, 57]. While the rRNA depletion approach captures both poly A+ and poly A- transcripts, the poly A+ selection method only captures transcripts with poly A tails. The results of a study by Zhao et al. [10] showed that just 6% of the reads from the poly A selection method were mapped to the intronic region, compared to about 50% of the reads from the rRNA depletion method. As a result, the poly A selection approach had substantially greater percentages of useable reads for gene quantification than the rRNA depletion method. However, numerous additional mRNAs without poly A tails are also excluded by poly A selection. The rRNA depletion strategy alone was able to identify new protein-coding genes, pseudogenes, short RNAs and lncRNAs (long non-coding RNAs) [10, 58]. For instance, the involvement of lncRNAs in the response of kiwifruit to *Pseudomonas syringae* pv. *actinidiae* infection has been revealed in a study [11]. Moreover, rRNA-depleted libraries are superior to poly A-selected libraries for degraded RNA samples [23, 59], and RNA-seq data from rRNA depletion offer novel insights into the transcriptional processes in cells [60]. Therefore, combining the two approaches (rRNA depletion and poly A selection) in dual RNA sequencing would be ideal to acquire deeper insights into the transcriptomic profiles of host-pathogen interactions because both selectively omit a diverse set of RNAs or target different fractions of the transcriptome. In this study, we have developed an effective dual RNA sequencing technique that uses both rRNA depletion and poly A selection to particularly enhance the bacterial mRNA in the mixed plant-bacterial samples. In comparison to the conventional method, the enriched method in our study using increased Dynabeads concentration (3.3X) has significantly improved the mapping ratio of *F. dauae* infected tomato, Xcv3 and Xag8ra infected peppers to the genome and CDS of their respective bacteria (Table 1; Fig. 2). Consistent with our study, Kumar et al. [61] have demonstrated that the bacterial mRNA enrichment method involving the concurrent removal of poly A and rRNA has tripled the amount of mRNA and the percentage of Wolbachia transcripts in a Wolbachia-Drosophila system.

While an increase in PCR cycles improved the mapping ratio of Xcv3 and Xag8ra infected peppers, it did not produce the necessary library length, as revealed by the size distribution pattern of cDNA libraries (Fig. S1). Hence it was confirmed that 10/12 PCR cycles was optimum for the ssRNA-seq library preparation using the enriched method. Upon examining different infection time points of Xcv3 in peppers, it was observed that the mapping ratio to the genome and CDS of Xcv3 increased as the time of infection increases (Fig. 3B). This finding corresponds with the fact that the amount of bacterial RNA in the infected plant tissue increases as time elapses leading to an increased pathogen mapping ratio. Similar to our finding, Li et al. [62] also observed a higher mapping ratio during compatible and incompatible interactions between potato and *Phytophthora infestans* at the later stage of infection (48 hpi). However, a significant correlation existed among the conventional and enriched methods in terms of gene expression during early stages of infection in 12 and 24 hpi (Fig. 3C). Consistent to this result, the number of DEGs were found greatly upregulated in early infection time points (Fig. 4). A significant upregulation of DEGs was obtained in the enrichment method at all examined fold change thresholds than that of the conventional method. The number of DEGs were maximum at 24 hpi in the enriched method irrespective of all fold change thresholds. The number of DEGs notably reduced at the later stage of infection (48 hpi) at both RNA-seq methods. This suggests that the host plant has displayed an effective defense response against the bacterial pathogen, Xcv3 during later stages of infection. Similar to our study, the number of upregulated DEGs were greatly reduced at the late stage of infection (60 hpi) by *Phytophthora nicotianae* in susceptible tobacco cultivar (XHJ) [63]. Likewise, in a *Brassica napus* line susceptible to stem rot causing fungal pathogen, *Sclerotinia sclerotiorum*, there were fewer number of differentially expressed genes at the late infection stages (24–48 hpi) compared to the early infection stages (8–16 hpi) [64]. Hence, our study employing the enriched method, proved to be effective in detecting a greater number of DEGs in the initial phases of infection, when bacterial RNA levels are relatively lower in comparison to the later stages of infection.

The GO analysis showed that, in both conventional and enriched approaches, particularly at 48 hpi, a considerable enrichment of DEGs occurred in activities linked to proteolysis, protein secretion by the type III secretion system (T3SS), kinases, serine-type endopeptidases, and heme binding (Fig. 5). Proteolysis serves multiple critical functions throughout the entire infection cycle of the pathogen, contributing significantly to its virulence mechanisms. Secreted proteases play a crucial role in promoting host penetration and effective dissemination by actively engaging in breaking down the host’s physical defenses [65]. Furthermore, proteolytic enzymes facilitate host colonization by counteracting the host’s defense mechanisms [66].

The T3SS is a common strategy used by many gram-negative bacteria to invade plants, including *Xanthomonas campestris* pv. *vesicatoria.* This system includes a translocon, a bacterial protein channel that integrates into the plasma membrane of the host and aids in the translocation of effector proteins into the host cell cytosol [67, 68]. The type III effectors are thought to impair a variety of host cellular processes, including cytoskeletal adjustments, vesicle transport, and defense reactions [69, 70]. The T3SS is encoded by a gene cluster known as *hrp* which are responsible for inflicting disease on susceptible plants while inducing the hypersensitive response, a rapid programmed cell death occurring at the site of infection on resistant plants [71, 72]. The genes involved in T3SS that encode *HrpA, HprB, HrpD* etc. exhibited elevated expression levels during *Pantoea stewartii* subsp. stewartii infection in corn plants leading to Stewart’s wilt disease [73]. Prior research using a luminous reporter demonstrated the active transcription of *hrp* genes encoding T3SS and its effectors during the late stages of infection with *Ralstonia solanacearum*, the causative agent of bacterial wilt in species of solanaceous plants [74]. Recently, a comprehensive investigation of *in planta* transcriptome of *R. solanacearum*, has demonstrated a significant upregulation of *hrp* genes and T3SS effectors in the xylem of asymptomatic/wilted potato plants [75].

Kinases are central players in the intricate signaling pathways involved in bacterial infections. They are involved in regulating both host responses to infection and bacterial virulence strategies. The hallmark of signaling pathways is the process of serine/threonine phosphorylation by kinases, followed by dephosphorylation by phosphatases [76]. In a previous study, the two component signaling pathway elements such as histidine kinases (HKs) and response regulators (RR) that regulate a mitogen-activated protein kinase cascade were found to be upregulated during the infection of *Fusarium oxysporum* causing vascular wilt disease in banana [77]. Many studies have documented that pathogens also modulate certain serine threonine protein kinases in host plants during infection. For instance Ca^2+^ dependent protein kinases (CDPKs) were actively upregulated in fungal induced defense responses in potato [78], hybrid poplar [79], and *B. napus* [80]. Serine proteases are endopeptidases which are involved in several key functions including cell signaling, protein metabolism, protein processing, blood coagulation and immunity regulation [81, 82]. Additionally, they are apparently found as a virulence factor in a variety of pathogenic bacteria. For instance, the primary virulence factor of the phytopathogenic bacterium *Clavibacter michiganensis*, which causes tomato wilting and canker, is a putative serine protease encoded by the *pat-1* gene [83]. In Gram-negative bacteria, iron plays a vital role in growth and colonization in host, as it acts as a coenzyme in fundamental cellular processes like cellular respiration and DNA synthesis [84]. During infection, the host immune response employs a strategy known as nutritional immunity to restrict the availability of free iron to the pathogens [85]. Although hosts have developed strategies to sequester iron to impede bacterial growth, pathogenic bacteria have simultaneously evolved mechanisms to evade these host defenses. Notably, Gram-negative pathogens possess outer membrane receptors designed to acquire ferrous iron and release siderophores and hemophores to capture iron-containing compounds such as heme or hemoglobin, facilitating their uptake into the bacterial cells [86, 87]. The enrichment of DEGs associated with heme binding activity in our study implies the significance of iron acquisition during the infection process of the pathogen. Therefore, the findings of our investigation strongly indicate that, proteolysis, secretion of effector proteins through T3SS, kinases, serine type endopeptidase and heme binding activities play a central role in the infection process of Xcv3 in peppers.

## Conclusions

The present study has demonstrated an efficient bacterial mRNA enrichment method for the dual RNA-seq involving the concomitant rRNA removal and poly A selection with increased Dynabeads concentration. In comparison to the conventional RNA-seq method, a significant improvement in the alignment of reads to the genomic as well as to the coding sequences of the pathogen was observed in the enriched method. At all fold change threshold levels studied at various stages of Xcv3 infection in peppers, the enriched method consistently showed a higher number of DEGs than the conventional method. Moreover, the enriched method was consistent with the conventional method in elucidating the GO terms with DEGs predominantly enriched in proteolysis, kinase, serine type endopeptidase and heme binding activities. Thus, the enriched method described in this study presents a novel and reliable approach to enhance the read mapping of bacterial counterparts in dual RNA-seq and decipher the complex transcriptomic changes involved in plant-bacterial interactions.

### Electronic supplementary material

Below is the link to the electronic supplementary material.

Supplementary Material 1

## Data Availability

The datasets supporting the conclusions of this article are included within the article or are available from corresponding author on reasonable request.
