# Supplementary material for: An improved bacterial mRNA enrichment strategy in dual RNA sequencing to unveil the dynamics of plant-bacterial interactions
Source: Plant Methods. 2024 Jul 1;20:99. doi: 10.1186/s13007-024-01227-x (PMC11218159; doi:10.1186/s13007-024-01227-x)
Supplement: Supplementary file 1 — Supplementary Material 1 [file 13007_2024_1227_MOESM1_ESM.docx]

**Supplementary Tables**

**Table S1**. List of primers used for real-time qRT-PCR analysis.

| **No.** | **Gene ID (ASM916v1)** | **Gene description** | **Forward (5’ 🡪 3')** | **Reverse (5’ 🡪 3')** | **Product size (bp)** |
| --- | --- | --- | --- | --- | --- |
| 1 | CAJ23004 | lepA | AAT ATC CGC AAC TTC TCC AT | TGA AAT TCA GGT GGT AGG TC | 205 |
| 2 | CAJ22057 | hrcU | TTC GAT TTT TCC GAA CAT ACA | GGA TTG AGG GAA TCG AAC TT | 194 |
| 3 | CAJ22060 | hrcJ | CAC GAT CAG GTG TCC TAT TC | CAT CGA TAT TCG ACA AGG TC | 181 |
| 4 | CAJ22069 | putative ISRSO12 transposase | ATG AAG AAG TCC CGT TTT AC | CTT GCG GTT TTC CTC TTC | 195 |
| 5 | CAJ24688 | extracellular serine protease | CTT CTT GAA TGG TTC CAA CG | CCA TCG ACT ATG GAA AGT GC | 171 |
| 6 | CAJ25348 | TonB-dependent outer membrane receptor | CCA CCA AAT TGA AAT CCA | CTG CAG TTG CAG GAA TTT | 200 |
| 7 | CAJ21932 | Hypothetical protein | CTA CCT ATC CCC AAG TGA CC | CGT TCT TGT TCG GTC ATA TC | 205 |

**Table S2**. Differential gene expression in Xcv3; Comparison of the number of upregulated and downregulated genes among conventional, enriched and both methods.

| Method | Time point | 12 hpi | | | 24 hpi | | | 48 hpi | | |
| --- | --- | --- | --- | --- | --- | --- | --- | --- | --- | --- |
|  | \|FC\| > standard | up | down | sum | up | down | sum | up | down | sum |
| Both | \|FC\| > 0.585 | 469 | 291 | 760 | 666 | 613 | 1279 | 967 | 919 | 1886 |
| Enriched |  | 159 | 135 | 294 | 138 | 135 | 273 | 77 | 96 | 173 |
| Conventional |  | 135 | 111 | 246 | 142 | 135 | 277 | 71 | 80 | 151 |
| Both | \|FC\| > 1 | 434 | 223 | 657 | 537 | 452 | 989 | 680 | 587 | 1267 |
| Enriched |  | 137 | 125 | 262 | 107 | 159 | 266 | 58 | 77 | 135 |
| Conventional |  | 120 | 113 | 233 | 91 | 101 | 192 | 54 | 53 | 107 |
| Both | \|FC\| > 1.585 | 336 | 71 | 407 | 374 | 206 | 580 | 418 | 264 | 682 |
| Enriched |  | 76 | 101 | 177 | 62 | 122 | 184 | 35 | 43 | 78 |
| Conventional |  | 66 | 63 | 129 | 34 | 58 | 92 | 35 | 28 | 63 |
| Both | \|FC\| > 2 | 266 | 31 | 297 | 288 | 111 | 399 | 328 | 157 | 485 |
| Enriched |  | 56 | 61 | 117 | 42 | 85 | 127 | 20 | 25 | 45 |
| Conventional |  | 41 | 35 | 76 | 24 | 28 | 52 | 15 | 24 | 39 |
| Both | \|FC\| > 2.322 | 229 | 9 | 238 | 234 | 59 | 293 | 277 | 102 | 379 |
| Enriched |  | 37 | 43 | 80 | 32 | 81 | 113 | 18 | 21 | 39 |
| Conventional |  | 29 | 17 | 46 | 19 | 13 | 32 | 8 | 12 | 20 |
| Both | \|FC\| > 2.58 | 203 | 4 | 207 | 211 | 30 | 241 | 243 | 72 | 315 |
| Enriched |  | 21 | 29 | 50 | 23 | 78 | 101 | 11 | 16 | 27 |
| Conventional |  | 23 | 15 | 38 | 16 | 7 | 23 | 7 | 6 | 13 |


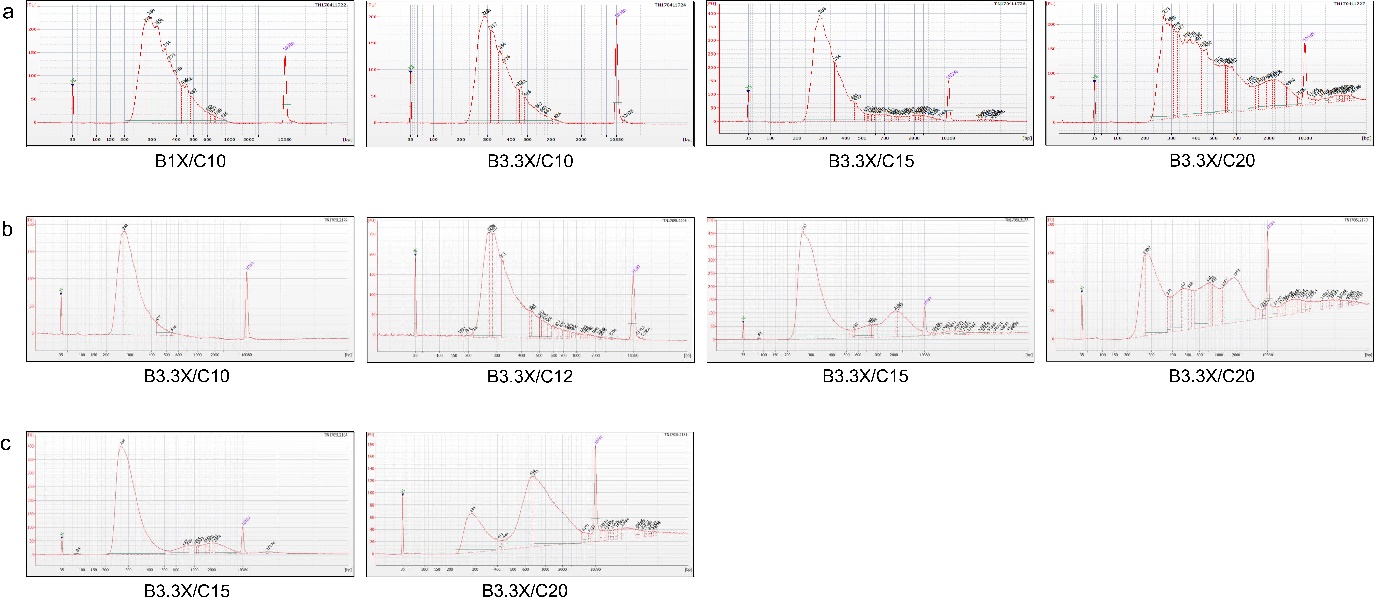


**Figure S1**. Size distribution of cDNA libraries according to various conditions of library preparation. (a) cDNA libraries from *F.* *dauae* infected tomato sample at 48 hpi. (b) cDNA libraries from Xcv3 infected pepper at 24 hpi. (c) Xag8ra infected pepper at 48 hpi. All libraries were validated by Bioanalyzer. The figures represent a technical replicate for each experimental condition. B, Dynabeads; 1X, the 1X volume of Dynabeads as the original protocol; 3.3X, the 3.3X volume of Dynabeads in the enriched protocol; C, number of PCR cycles in PCR enrichment step.


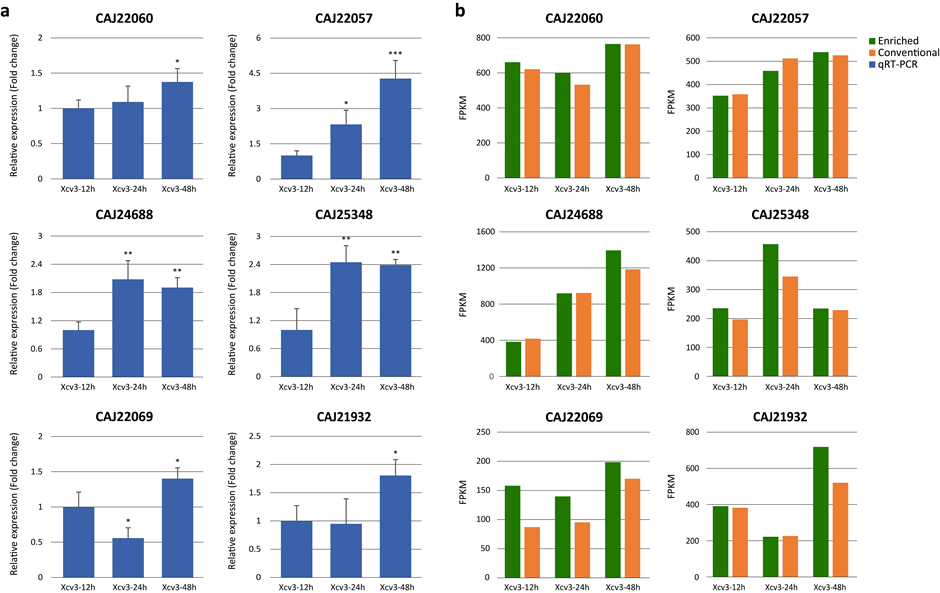


**Figure S2**. The validation of dual RNA-seq results through qRT-PCR analysis. (a) Represents qRT-PCR results of six genes whose expression was significantly changed by DEG analysis. To standardize the qRT-PCR data, reference genes from *lepA* were used. Statistically significant differences compared with the Xcv3 infected pepper at 12 hpi, determined using Student’s t-test are indicated by asterisks (* *p* < 0.05; ** *p* < 0.01, *** *p* < 0.001). (b) Represents the results of dual RNA-seq quantification of 6 genes normalized to FPKM.
